# Mediating Effects of Serum Lipids and Physical Activity on Hypertension Management of Urban Elderly Residents in China

**DOI:** 10.3390/metabo14120707

**Published:** 2024-12-15

**Authors:** Yang Zhao, Yike Zhang, Fei Wang

**Affiliations:** 1 Sports Science Institute, Shanxi University, Taiyuan 030006, China; zhaoyang@sxu.edu.cn (Y.Z.);; 2 School of Physical Education, Shanxi University, Taiyuan 030006, China

**Keywords:** serum lipids, physical activity, SEM, hypertension management, urban elderly residents, China

## Abstract

**Background/Objectives**: Investigating the importance and potential causal effects of serum lipid biomarkers in the management of hypertension is vital, as these factors positively impact the prevention and control of cardiovascular disease (CVD). **Methods**: We surveyed 3373 urban residents using longitudinal data from the CHARLS database, collected between 2015 and 2020. Pearson correlation methods were employed to explore the relationships among the numerical variables. A logistic regression model was utilized to identify the risk factors for hypertension. The dose–effect relationship between serum lipids and BP was assessed using restricted cubic splines (RCS). Additionally, piecewise structural equation modeling (PiecewiseSEM) was conducted to further elucidate the direct and indirect pathways involving individual body indices, serum lipids, and PA on BP responses at different levels of physical activity (PA). **Results**: The four serum lipids showed significant differences between hypertensive and non-hypertensive residents (*p* < 0.05). All lipids, except for HDL cholesterol, demonstrated extremely significant positive correlations with both systolic blood pressure (SBP) and diastolic blood pressure (DBP) (*p* < 0.001). All serum lipid variables were significantly associated with the incidence of hypertension. Specifically, triglycerides (bl_tg), HDL (bl_hdl), and low-density lipoprotein LDL cholesterol were identified as significant risk factors, with odds ratios (ORs) of 1.56 (95% CI: 1.33–1.85, *p* < 0.001), 1.16 (95% CI: 1.02–1.33, *p* < 0.05), and 1.62 (95% CI: 1.23–2.15, *p* < 0.001), respectively. Conversely, cholesterol (bl_cho) was a protective factor for hypertension, with an OR of 0.60 (95% CI: 0.42–0.82, *p* < 0.01). PA showed weak relationships with blood pressure (BP); however, PA levels had significant effects, particularly at low PA levels. The four serum lipids had the most mediating effect on BP, especially under low PA level conditions, while PA exhibited a partly weak mediating effect on BP, particularly under high PA level conditions. **Conclusions**: Serum lipids have significant nonlinear relationships with BP and PA levels exert different influences on BP. The significant mediating effects of serum lipids and the weak mediating effects of PA on individual body indices related to SBP and DBP demonstrate significant differences across varying levels of PA, highlighting the importance of low PA levels in hypertension management. This study could provide valuable recommendations and guidance in these areas.

## 1. Introduction

As society has evolved, the lifestyles of urban residents have shifted significantly, leading to a high prevalence of chronic diseases [1]. Hypertension, defined as a systolic blood pressure (SBP) of 140 mmHg or higher and/or a diastolic blood pressure (DBP) of 90 mmHg or higher, is a prevalent condition that affects a significant portion of the population and is a major risk factor for cardiovascular disease [2]. The prevalence of hypertension in China has increased from 5.1% to 23.2% over the past three decades [3,4], and the number rose to 56.8% in northeast China in the 2020s [5]. The number of hypertensive patients aged 30 to 79 years has doubled from 1990 to 2019 [6] and is projected to reach 1.56 billion by 2025 [7]. Meanwhile, the number of fatalities caused by hypertension among Chinese residents was 2.54 million in 2017, with cardiovascular disease deaths accounting for 95.7% [8]. Hypertension has become a significant public health concern worldwide. Studies have shown that it is the leading risk factor contributing to the global burden of disease [9]. In China, hypertension sharply increases with age and is a significant risk factor for ischemic heart disease, stroke, chronic kidney disease, and dementia [4]. It imposes a heavy burden on families and society.

Due to common risk factors or disease pathogenesis, hypertension often coexists with other chronic diseases, such as dyslipidemia [10]. The results of a 2018 national survey indicated that the prevalence of dyslipidemia among adults over 18 years old was 35.6% [11]. Hypertension and dyslipidemia are the most common risk factors for cardiovascular disease (CVD) [12]. The coexistence of hypertension and dyslipidemia significantly increases the harmful effects on the cardiovascular system [13]. The result of this interaction is an exponential increase in CVD. Moreover, controlling BP and serum lipids has become a priority for the prevention and management of CVD. Hypertension, dyslipidemia, and obesity are independent risk factors for CVD, and they often co-exist [12]. These conditions represent a serious medical issue that significantly increases the risk of heart, brain, and other diseases. In China, 23.2% of adults aged 18 and above have hypertension [3]. Dyslipidemia is recognized as an independent risk factor for atherosclerosis, coronary heart disease, and stroke. During three national cross-sectional surveys conducted from 2002 to 2015, serum lipid levels increased sequentially among Chinese adults [4].

Physical activity (PA) has proven to be an effective tool for the primary and secondary prevention of several chronic diseases [14]. The intensity of PA also plays a role, as moderate- to high-intensity activities have been shown to significantly reduce BP in hypertensive patients and lower the risk of CVD [15,16,17]. A study of 1311 African Americans found that exercise-related PA had the potential to lower the risk of developing high BP, while other types of activity were not associated with this risk [18]. Another investigation involving 125,402 adults found that longer commuting times and high-intensity leisure activities were significantly associated with a lower risk of hypertension, especially among older individuals [19]. Most previous studies have examined the effects of PA on BP. However, current global estimates indicate that one in four adults and 81% of adolescents do not engage in the recommended levels of PA as suggested by the WHO [20]. In China, PA was observed to be in sharp decline while urbanization was increasing dramatically [21]. Additionally, some studies have examined the impact of age, gender, waist circumference, and BMI on the development of hypertension, revealing a positive correlation between these factors and BP [22]. The study by Wang et al. [23] revealed the significant impact of body shape control on managing hypertension. All these variables were incorporated into the statistical modeling to explore their impact on BP. While PA has beneficial effects on the chronic diseases mentioned above, the mechanism by which PA influences BP through serum lipid metabolites remains unclear. Furthermore, the sequence of effects between PA and serum lipids in managing hypertension has not been reported. Investigating the significance of serum lipid biomarkers in the context of PA for hypertension management is essential for lifestyle interventions aimed at preventing cardiovascular disease (CVD) and improving overall health. Additionally, understanding the pathways through which PA and blood lipids influence CVD risk factors can enhance prevention and disease control strategies. Mediation analysis is commonly used to explore the mechanisms of interventions, and epidemiologists have increasingly employed it to analyze mechanisms focused on path analysis in recent years. However, studies examining the mechanisms by which PA and serum lipids affect BP are limited, particularly concerning the internal pathway effects among different constructs. The objective of the study is (1) to clarify the relationship between BP and PA, serum lipids, and other relevant variables; (2) to quantify the effects of PA and serum lipids on BP; and (3) to assess the underlying mechanisms among PA, serum lipids, and BP. This study aims to provide theoretical support for research on hypertension in elderly urban residents and offer valuable insights into hypertension management.

## 2. Materials and Methods

In this study, we selected residents from the China Health and Retirement Longitudinal Study (CHARLS) databases from 2015 to 2020. It is a longitudinal database that began in 2008, with individuals followed up every two years. All data will be made public one year after the completion of data collection. In our study, the updated database extends to 2020, and recently, new data delayed by COVID-19 have been published. The information collected by CHARLS includes highly scientific and standardized health and medical measurements and provides valuable data on social and economic environments. These data have demonstrated significant contributions to research. Detailed information regarding the study design and sampling strategy can be found in the cohort information [24]. In the data extraction process, we first extracted variables including height, weight, age, gender, smoking and drinking habits, urban/rural residency, waist circumference, sleep duration, PA, and four serum lipids. We then filtered the data to include only individuals with systolic blood pressure (SBP) ≥ 140 mmHg, and/or diastolic blood pressure (DBP) ≥ 90 mmHg, and/or those using blood pressure medication, as well as individuals aged over 60. Finally, we removed any missing values for these variables, resulting in a final sample of 3373 elderly residents aged 60 years and older. All participants provided informed consent, and CHARLS was approved by the Institutional Review Board of Peking University (Code: IRB00001052-11015).

The serum lipid data included triglycerides (bl-tg), high-density lipoprotein cholesterol (bl-hdl), low-density lipoprotein cholesterol (bl-ldl), and total cholesterol (bl-tc). The final sample comprised 1649 individuals with hypertension and 1724 without hypertension. Among these respondents, there were 2663 females and 710 males.

Metabolic equivalent (MET) was used to calculate PA intensity.

(1) $$\mathrm{PA}\;=\sum_{i}\left({\mathrm{MET}}_{i}\times {\;F}_{i}\times {\;T}_{i}\right)$$

where F*_i_* is the weekly frequency of activity of the PA level (day/week); T*_i_* is the activity time of day on the PA level (minutes/day); MET*_i_* is the constant related to the PA level. The PA levels are divided into three types: vigorous PA (VPA), moderate PA (MPA), and light PA (LPA). According to the Chinese Guidelines of the International Physical Activity Questionnaire (IPAQ) [25], the MET for VPA, MPA, and LPA were assigned 8.0, 4.0, and 3.3, respectively. The PA duration is categorized as follows: “1 = (<0.5 h), 2 = (0.5 h ~ 2 h), 3 = (2 h ~ 4 h), 4 = (≥4 h)” [26]. In this study, PA intensity was ultimately categorized into three levels according to the recommendation from the World Health Organization (WHO) [27,28]: Low (≤10 MET·h/week), Moderate (10 ~ 50 MET·h/week), and High (≥50 MET·h/week) levels.

The demographic data of participants are given in Table 1. Continuous numeric variables are presented as the mean ± standard deviation, while categorical variables are presented as frequency and percentage. Spearman correlation analysis was used to determine the relationship between BP and all other numeric variables. A logistic regression model was employed to identify protective and risk factors for hypertension. The dose–response effects of serum lipids and BP were evaluated using restricted cubic splines (RCS) for non-linear correlations and piecewise linear regression to identify inflection points, considering significance (*p* < 0.05). We used the piecewiseSEM R package for structural equation modeling (SEM) to further assess the associations among serum lipids (bl_tg, bl_hdl, bl_ldl, and bl_cho), PA, and individual body indices (height, weight, and waist) affecting SBP and DBP, respectively, after accounting for multiple key factors such as age and sleep time. In SEM, we used path analysis to explore potential causal relationships between variables. These relationships are represented by line segments, where an arrow pointing to a variable indicates that another variable influences it, and the number on the line represents the path coefficient. All measured variables included in this model were first standardized and then included in the SEM. To confirm the robustness of the relationships between serum lipids and PA, we used piecewiseSEM to account for random effects related to hypertension, providing the “marginal” and “conditional” contributions of serum lipids and PA predictors in driving model stability.

All statistical analyses and diagrams were conducted using R (version 4.2.1, https://cran.r-project.org, accessed on 1 July 2022). In the data cleaning process, the timestamp was handled using the “janitor” and “lubridate” packages in R. The “tidymodels” package was used for data cleaning and preprocessing of all predictors. Descriptive analysis was performed using the “tbl_summary” package, while Pearson’s correlation analysis utilized the “corr” package. Logistic regression was carried out using the “log” package. The RCS analysis was conducted using the “ggrcs” package. The SEM analyses were performed using the “piecewiseSEM,” “nlme,” and “lme4” packages. We used Fisher’s C test (when 0.05 < *p* < 1.00) to confirm the goodness of fit of the modeling results. We then modified our models based on significance (*p* < 0.05) and the overall goodness of fit. All plots were generated using the “ggplot2” package.

## 3. Results

### 3.1. Descriptive Statistics

Residents with hypertension exhibited significant differences from non-hypertensive residents in individual categorical variables and most continuous variables (Table 1). Among the continuous variables, four serum lipid levels showed significant differences between hypertensive and non-hypertensive residents (*p* < 0.05). Except for bl-hdl, higher values were observed in hypertensive residents. PA showed relatively higher in hypertensive residents, though differences in total PA absolute values were not statistically significant compared to non-hypertensive residents. However, significant differences were found across different PA levels (*p* < 0.05). In terms of continuous variables, the waist circumference of hypertensive patients was measured at 88.54 ± 13.51 cm, significantly higher than that of non-hypertensive patients, which was 83.30 ± 25.85 cm (*p* < 0.001). Additionally, other variables such as weight (59.43 ± 12.22 kg for hypertensive patients compared to 55.24 ± 25.01 kg for non-hypertensive patients) and age (68.42 ± 6.40 years for hypertensive patients compared to 66.74 ± 5.93 years for non-hypertensive patients) were also higher in hypertensive patients. Both height and sleep duration showed no significant differences between the two groups (*p* > 0.05). Furthermore, among the categorical variables, only the residency status (urban vs. rural) exhibited significant differences (*p* < 0.001) between hypertensive and non-hypertensive residents.

### 3.2. Pearson Correlation Analysis

**Figure 1:**
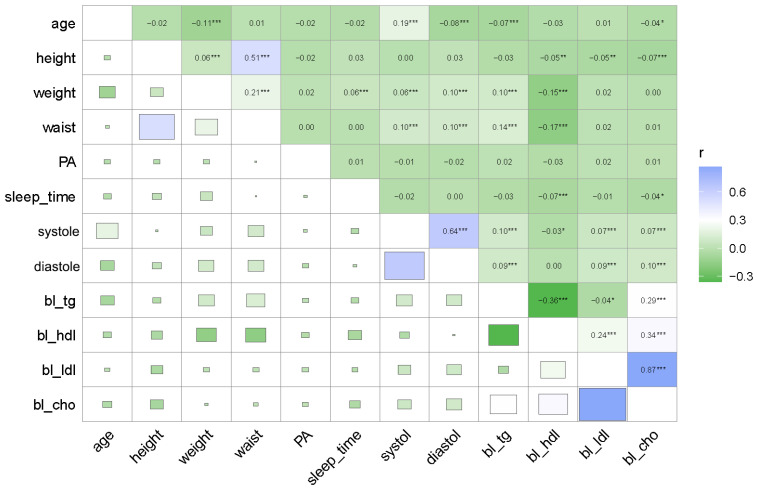
Spearman correlation analysis of four serum lipids variables, PA, SBP, DBP, and various numerical variables: The numbers represent correlation coefficients, and markers represent significance levels as * *p* < 0.05, ** *p* < 0.01, and *** *p* < 0.001.

The Pearson correlation analysis was used to determine the relationships among PA, four serum lipids variables, SBP, DBP, and various numerical variables (Figure 1). As shown in Figure 1, age, waist circumference, weight, and four serum lipids—except for high-density lipoprotein cholesterol (bl-hdl)—demonstrated extremely significant positive correlations with both SBP and DBP (*p* < 0.001). The serum lipid of bl-hdl showed a significant negative correlation with SBP (*p* < 0.05). Additionally, bl-hdl, triglycerides (bl-tg), and age exhibited more significant correlations with other numerical variables. Sleep time only showed significant correlations with two serum lipids: bl-hdl and total cholesterol (bl-cho).

### 3.3. Logistic Regression Analysis

**Figure 2:**
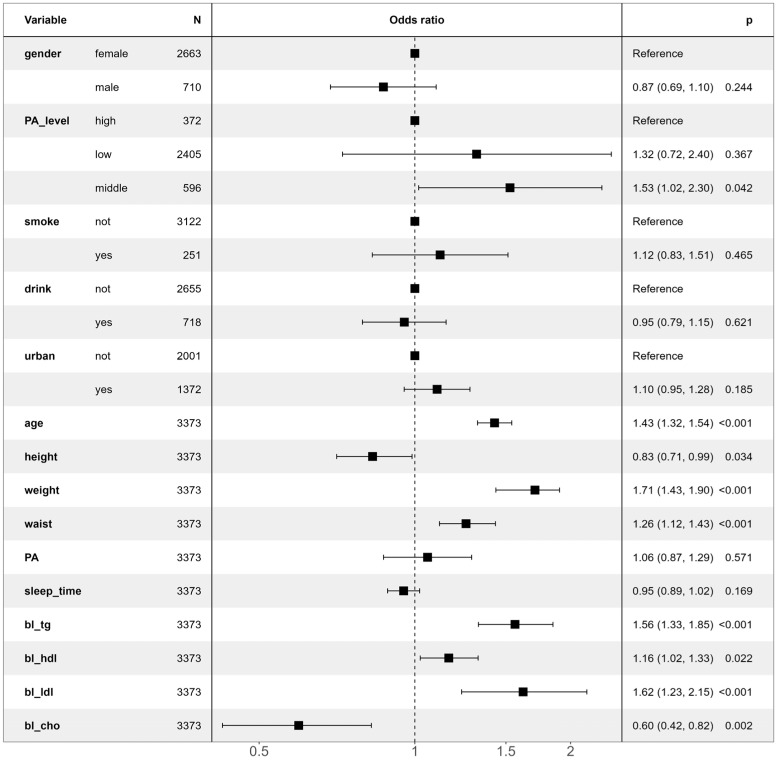
Factors affecting hypertension based on logical regression model.

Residents, both with and without hypertension, were included as a binary dependent variable in the study. Binary logistic regression analysis was conducted to identify potential risk factors for hypertension. Figure 2 illustrates the significant influencing factors on hypertension based on the logistic regression model.

The results indicated that three serum lipids—bl_ldl (OR = 1.62, 95% CI: 1.23–2.15, *p* < 0.001), bl_tg (OR = 1.56, 95% CI: 1.33–1.85, *p* < 0.001), and bl_hdl (OR = 1.16, 95% CI: 1.02–1.33, *p* < 0.05)—were potential risk factors for hypertension. Additionally, one serum lipid, bl_cho (OR = 0.60, 95% CI: 0.42–0.82, *p* < 0.01), was identified as a potential protective factor. Moreover, numeric variables such as age, waist circumference, and weight were also significant risk factors for hypertension.

In terms of categorical variables, having a moderate PA level compared to a high PA level was identified as a risk factor for hypertension (OR = 1.53, 95% CI: 1.02–2.30, *p* < 0.05), while other categorical variables were not identified by the logistic regression model. The result indicates that, compared to residents with high PA levels, those with moderate PA levels faced a 53% increased risk of developing hypertension. Conversely, the numerical variable of height was found to be a slight protective factor against hypertension (OR = 0.83, 95% CI: 0.71–0.99, *p* < 0.05).

### 3.4. Dose–Effects of Serum Lipids with BP

**Figure 3:**
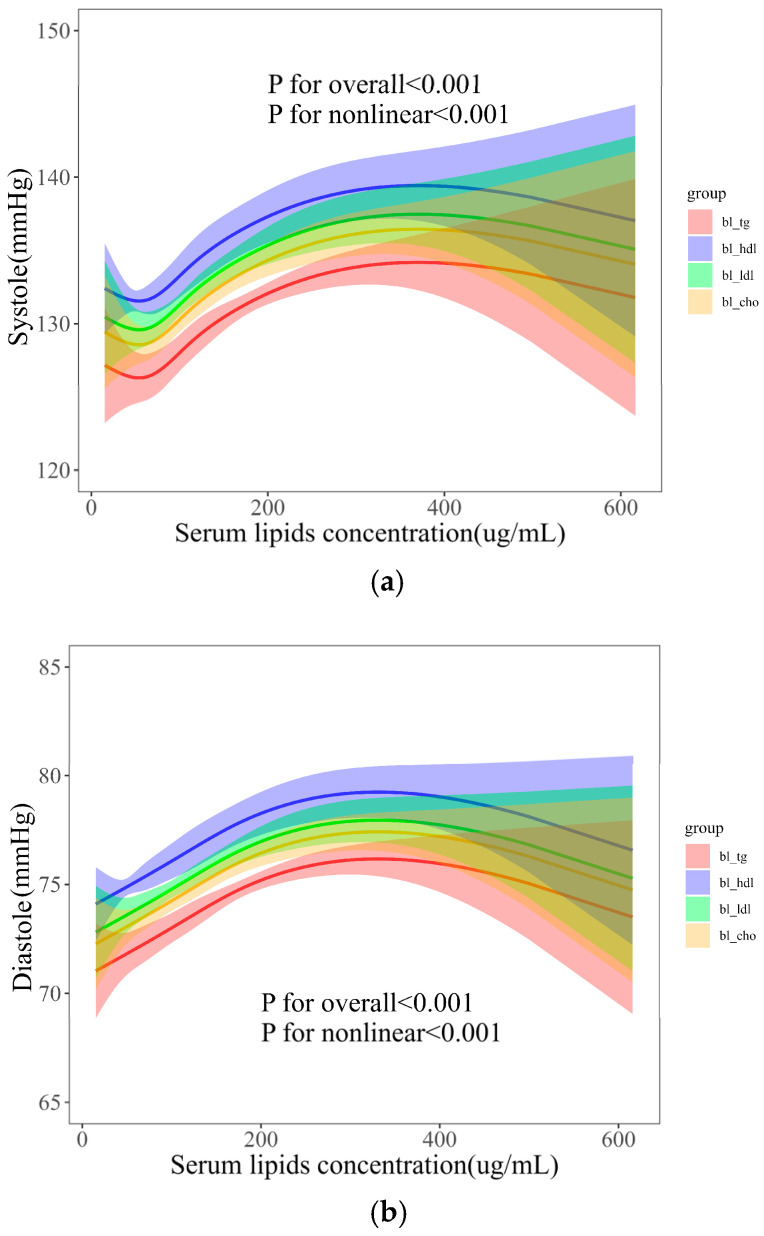
Dose–effects of serum lipids on systole (**a**) and diastole (**b**).

Our analysis initially indicated a potential non-linear relationship between serum lipids and both SBP and DBP. Utilizing RCS analysis, we identified a non-linear relationship between serum lipids and BP (Figure 3a for SBP and Figure 3b for DBP). The breakpoint that defines the natural spline was 4. In the RCS model, we adjusted the covariables including age, waist circumference, height, weight, sleep time, and PA. Age showed a highly significant positive effect on both SBP and DBP (*p* < 0.001). However, sleep time showed a significant negative effect on SBP (*p* < 0.05). The baseline body indices of height, weight, and waist circumference also demonstrated highly significant positive effects on both SBP and DBP (*p* < 0.001). In addition, the effects of serum lipids showed similar trends for both SBP and DBP, with the highest effects observed for baseline high-density lipoprotein (bl_hdl), followed by baseline low-density lipoprotein (bl_ldl), baseline cholesterol (bl_cho), and baseline triglycerides (bl_tg). This suggests that the relationship between serum lipids and both SBP and DBP is not simply linear but exhibits an inverted U-shaped relationship.

### 3.5. Mediating Effects of PA and Serum Lipids on BP

**Figure 4:**
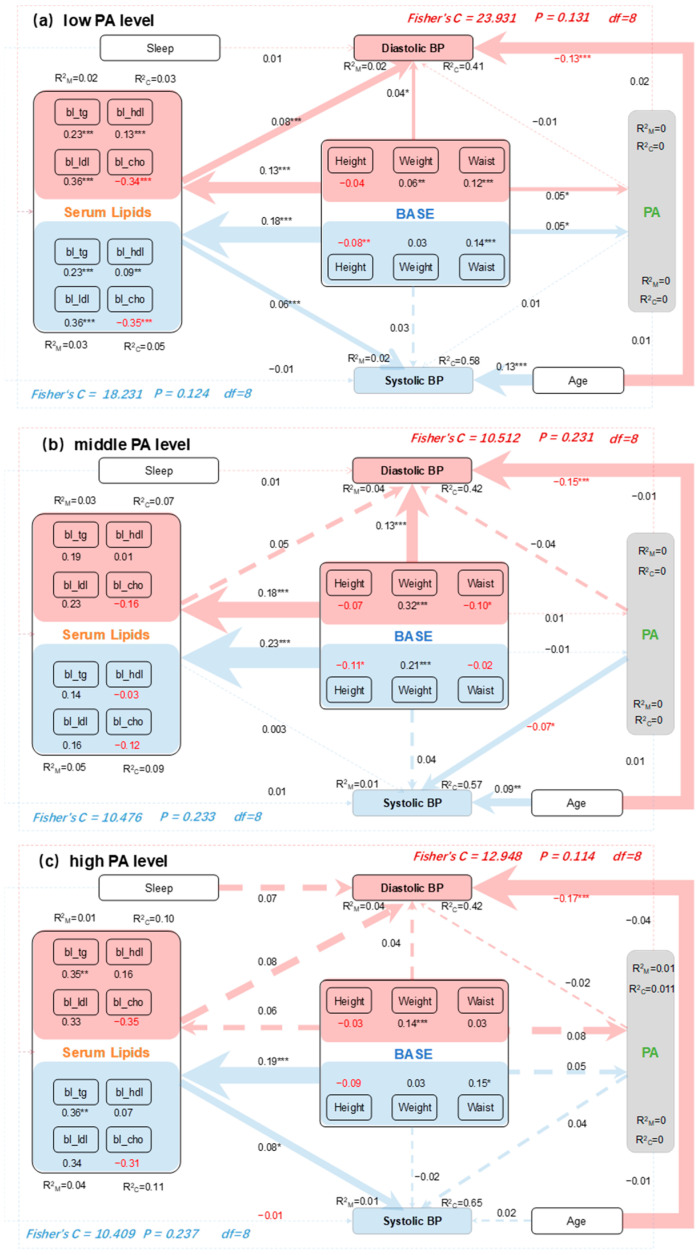
Piecewise SEM accounts for the direct and indirect effects of individual base, serum lipids, physical activity (PA), age, and sleep time on SBP (blue color) and DBP (red color) under low PA levels (**a**), moderate PA levels (**b**), and high PA levels (**c**), respectively. The individual base and serum lipids were combined into composite variables. Numbers adjacent to the measured variables represent their coefficients with the composite variables after the data were standardized. Numbers next to the arrows indicate path coefficients, which reflect the directly standardized effect size of the relationships. The thickness of the arrows represents the strength of these relationships. The total standardized effects of composite variables on blood pressure (BP) are shown in the marginal and conditional R^2^, representing the proportion of variance explained by all predictors without (R^2^m) and with (R^2^c) accounting for random effects of “Hypertension?”. Relationships between residual variables of measured predictors are not shown. Significance levels for each predictor are indicated as * *p* < 0.05, ** *p* < 0.01, and *** *p* < 0.001.

Piecewise SEM was performed to uncover the direct and indirect pathways through which individual baseline characteristics, serum lipids, PA, age, and sleep duration have effects on blood pressure (BP) responses at different PA levels. The direct and indirect pathways by which these regulatory factors impact variations in SBP and DBP are illustrated in Figure 4. The combined influence of individual baseline characteristics, serum lipids, PA, age, and sleep duration accounted for a significant proportion of SBP variations at low PA levels (58%), moderate PA levels (57%), and high PA levels (65%), considering the random effects of “Hypertension?” (blue sections of Figure 4). Similarly, these variables explained a substantial proportion of DBP variations at low PA levels (41%), moderate PA levels (42%), and high PA levels (42%), again considering the random effects of “Hypertension?” (red sections of Figure 4).

Aside from age, individual baseline variables—including height, weight, and waist circumference—consistently played a significant role in reflecting the regulations on both SBP and DBP, both directly and indirectly, by exerting influences on serum lipids and PA. Among these baseline variables, height and waist circumference were the most important factors affecting SBP (blue sections in Figure 4a,c) and DBP (red sections in Figure 4a,b) in response to changes in serum lipids. Weight significantly had impacts on serum lipids related to SBP, with HDL cholesterol (bl_hdl) being the most influential, particularly by reducing the effects at moderate PA levels (blue section in Figure 4b). Conversely, waist circumference had a strong impact on four serum lipids related to DBP by enhancing the effects at low PA levels (red section in Figure 4a). Furthermore, in both low and high PA level conditions, the mediating effects of serum lipids were particularly important for SBP (blue sections in Figure 4a,c), especially concerning triglycerides (bl_tg). Similarly, in low PA conditions, the mediating effects of serum lipids were also significant for DBP (red section in Figure 4a). Additionally, PA demonstrated only a weak mediating effect on DBP, particularly under low PA conditions (red section in Figure 4a). Moreover, we observed direct effects of serum lipids and PA on both SBP and DBP, which may also lead to indirect impacts from individual baseline variables across different PA levels (Figure 4).

## 4. Discussion

A lack of sufficient PA can negatively impact health in daily life, contributing to many chronic diseases, especially in the prevention of chronic diseases [17]. Our research revealed that PA levels could lead to significant differences in hypertension, based on ANOVA and logistic regression models. One previous meta-analysis found that individuals who engaged in moderate-intensity PA experienced an 18% reduction in the risk of developing hypertension [29]. A randomized clinical trial demonstrated that participants in the exercise group, who followed a 12-week aerobic program, experienced significant reductions in their 24 h ambulatory SBP and DBP [30]. These studies support our findings. There is already ample evidence confirming the effectiveness of sensible exercise in controlling hypertension. It is clear that appropriate forms of exercise and exercise intensity can contribute to the management and control of BP. Moreover, our findings suggest that achieving certain levels of PA intensity may be particularly important for hypertension management.

Serum lipids have been recognized as independent risk factors for atherosclerosis, coronary heart disease, and stroke. Our findings demonstrated that the four serum lipids are highly correlated with SBP and DBP, as well as with individual baseline variables. Among the continuous variables related to the risk of developing hypertension, we found that weight, age, and serum lipid-related variables were significantly associated with hypertension development (*p* < 0.05). Each unit increase in age and weight was associated with 1.43 times (OR = 1.43, 95% CI: 1.32–1.54, *p* < 0.001) and 1.84 times (OR = 1.84, 95% CI: 1.43–1.90, *p* < 0.001) increase in the risk of hypertension, respectively. These results are consistent with previous studies. The research by He et al. revealed that higher bl_tg and lower bl_hdl increased the risk of new-onset hypertension [31]. As age increases, blood vessels undergo degenerative changes, such as a decline in wall elasticity [32] and a deterioration in permeability [33], which prevents vessels from expanding and contracting efficiently, leading to increased BP. Previous research also indicated that several anthropometric indices show a significant correlation with hypertension. These indices can be included in an individual’s medical history and used as tools for cardiovascular health screening, potentially yielding better results for public health [34]. These physiological processes related to aging are generally irreversible. Although age is an immutable risk factor for hypertension, adopting a healthy lifestyle may help minimize the risk of developing the condition. Our study also identified weight management as a key factor in preventing and controlling hypertension. A survey of Korean adolescents indicated that overweight and obese individuals had 1.52 and 1.89 times the risk of hypertension and exhibited a high clustering of cardiometabolic risks [35]. A study involving a Chinese population of middle-aged and elderly individuals revealed that all 14 indicators of obesity and adiposity were risk factors for hypertension [36]. Increased visceral lipids worsen lipid profiles and promote abdominal fat deposition, leading to inflammation and oxidative stress associated with obesity, atherosclerosis, and hypertension. These factors disrupt insulin metabolism and renal sodium retention, further affecting BP [36,37]. Our results revealed that all serum lipid variables were significantly associated with the incidence of hypertension. Specifically, triglycerides, high-density lipoprotein, and low-density lipoprotein were significant risk factors. In contrast, cholesterol was identified as a protective factor for hypertension. The mean cholesterol levels in both hypertensive and non-hypertensive elderly residents were within the recommended safe range (<200 mg/dL) in this study, which may explain the reason.

In addition, the impact of waist circumference on BP cannot be ignored. Evidence suggests that increased waist circumference elevates the risk of hypertension in older individuals [22]. Wang et al. analyzed data from a survey involving 5742 men and 5972 women, finding that waist circumference could potentially raise BP levels [23]. These studies highlight the significant influence of factors such as weight and waist circumference on BP. Our descriptive statistics demonstrate that hypertensive patients generally have higher values for all body morphology variables compared to non-hypertensive individuals, further underscoring the strong association between obesity and hypertension. The link between sleep and BP should also not be overlooked [27].

The underlying mechanisms of the relationship between obesity and hypertension are not fully understood. According to our SEM results, serum lipids exert the most significant mediating effect on BP, particularly under low PA conditions, while PA has a weaker mediating effect on BP, especially under high PA conditions. Additionally, height and waist circumference or weight are the most important variables influencing BP in response to increased serum lipids. Notably, high-density lipoprotein showed the most significant mediating effect in reducing the impact of weight on SBP under moderate PA conditions. It is well-known that high-density lipoprotein serves as the main apolipoprotein in the human body, promoting the synthesis of free cholesterol accumulated in peripheral tissues and lipoproteins in the bloodstream. It transports cholesterol to the liver for metabolism, maintaining lipid balance. As adipose tissue increases, HDL consumption rises, leading to the accumulation of unmetabolized neutral fats and lipid substances, which increases blood viscosity and contributes to dyslipidemia, thereby elevating blood pressure [13]. The relationship between high-density lipoprotein and BP is complex and varies under different conditions, indicating that more mechanistic studies at the cellular and molecular levels are needed to clarify this connection. Under both low and high PA conditions, triglycerides showed significant mediating effects on SBP. These findings suggest that serum lipids influence BP both directly and indirectly, while also highlighting the role of PA levels. Moreover, these proofs elucidate the associations among serum lipids, PA, and the primary CVD risk factors in elderly adults. They also focus on the internal relationships when PA and serum lipids do not show direct correlations and explore possible reasons through mediation effect analysis. Previous studies have indicated a positive association between weight and serum triglycerides [11], which is consistent with our findings, notably in relation to DBP under low PA conditions.

Our results suggest that increased lipid release from the body into the bloodstream under low PA conditions regulates metabolic pathways and influences BP. Although our findings did not show significant mediating effects of PA, some research indicates that serum lipids may act as moderators of PA [38]. The main reason for this could be the confounders adjusted in the model. We adjusted for all potential variables and random factors that may have contributed to the weak mediating effects of PA. Our results indicate that reducing adipose tissue is an important strategy for preventing and controlling hypertension at low PA levels. Additionally, increasing PA levels may affect blood lipid levels, and elevated fat could lead to hyperinsulinemia, disorders of the renin–angiotensin–aldosterone system, and ultimately vasoconstriction [39]. Previous studies have noted that dyslipidemia is closely related to hypertension [13]. This relationship is primarily reflected in excessive dietary intake of saturated fatty acids and a lack of PA, leading to increased blood viscosity, thickening of blood vessel walls, heightened mobility of vascular, and ultimately vascular lumen stenosis, reduced blood flow speed, and increased vascular pressure [23,39].

Based on mechanisms identified in previous studies, it is suggested that PA can influence BP. Furthermore, the impact of PA on the hypertension health of urban residents should not be ignored. Selecting appropriate intensity and types of PA can significantly affect BP levels. Although the results were verified through statistical analysis, this study has its limitations. The analysis of mediation variables may be insufficient. We adjusted for as many confounders as possible, and the estimated values were reasonable and strictly controlled. As more confounders are controlled, the stability of the relationships among independent variables, dependent variables, and mediating variables becomes contingent on sample size and data structure, necessitating the application of a multilevel mediation effect model. The anthropometric methods used to measure PA were not the most precise available, and a significant proportion of participants were excluded from our analysis. Therefore, generalizing our results to the entire survey population should be performed with caution. Furthermore, the current study has several limitations. The PA data came from self-reported questionnaires, and there may be some bias. The attrition of participants and the replenishment of queues are inevitable and may affect the results of the analysis. In future studies, we could consider additional variables and construct more reasonable frameworks using a multiple-level moderated mediation effect model to reveal more robust associations. Meanwhile, other microsystem indicators, such as metabolism-related indicators, hypertension-related genes or expressed proteins, and other molecular indicators, may effectively address the management and prevention of hypertension at various exercise intensities.

## 5. Conclusions

(1) Serum lipids have significant nonlinear relationships with BP, and PA levels have varying effects on BP. Meanwhile, the BP health of urban residents is influenced by factors such as age, weight, and waist measurements.
(2) There are significant mediating effects of serum lipids and weak mediating effects of PA on the relationship between individual body variables and SBP/DBP. These effects differ among PA levels, highlighting the importance of low PA levels in elderly hypertension management.

## Figures and Tables

**Table 1:** Statistical tables of the baseline characteristics of the population.

Characteristic [*n*/N(%)|Mean (SD)] ^1^	Unit	Hypertension?		*p*-Value ^2^
		not, N = 1724	yes, N = 1649	
Gender				0.606
Male	—	369/1724 (21%)	341/1649 (21%)	
Female	—	1355/1724 (79%)	1308/1649 (79%)	
PA_level				0.015
Low	—	1248/1724 (72%)	1157/1649 (70%)	
Moderate	—	274/1724 (16%)	322/1649 (20%)	
High	—	202/1724 (12%)	170/1649 (10%)	
Smoke				0.764
Yes	—	126/1724 (7.3%)	125/1649 (7.6%)	
Not	—	1598/1724 (93%)	1524/1649 (92%)	
Drink	—			0.206
Yes	—	382/1724 (22%)	336/1649 (20%)	
Not	—	1342/1724 (78%)	1313/1649 (80%)	
Urban				<0.001
Yes	—	648/1724 (38%)	724/1649 (44%)	
Not	—	1076/1724 (62%)	925/1649 (56%)	
Age	years	66.74 (5.93)	68.42 (6.40)	<0.001
Height	cm	154.00 (22.27)	153.81 (22.93)	0.465
Weight	kg	55.24 (25.01)	59.43 (12.22)	<0.001
Waist	cm	83.30 (25.85)	88.54 (13.51)	<0.001
PA	MET·h/w	15.62 (29.92)	16.20 (28.95)	0.223
Sleep_time	h	6.19 (2.09)	6.08 (2.14)	0.116
Systole	mmHg	119.35 (11.92)	146.42 (18.85)	<0.001
Diastole	mmHg	69.27 (8.09)	80.29 (11.21)	<0.001
bl_tg	μg/mL	131.88 (80.28)	155.38 (90.09)	<0.001
bl_hdl	μg/mL	53.11 (11.37)	51.17 (11.10)	<0.001
bl_ldl	μg/mL	105.63 (27.86)	107.78 (29.44)	0.023
bl_cho	μg/mL	188.72 (36.11)	192.14 (36.38)	0.004

Notes: ^1^ Mean (SD) for numeric variables; n/N (%) for categorical variables; ^2^ Pearson’s Chi-squared test for categorical variables; Kruskal–Wallis rank sum test for numeric variables.

## Data Availability

The data and R codes that support the findings of this study are available on request from the corresponding author.
